# Prediction of metabolic syndrome among postmenopausal Ghanaian women using obesity and atherogenic markers

**DOI:** 10.1186/1476-511X-11-101

**Published:** 2012-08-10

**Authors:** Fareed K N Arthur, Michael Adu-Frimpong, James Osei-Yeboah, Faustina O Mensah, Lawrence Owusu

**Affiliations:** 1 Department of Biochemistry and Biotechnology, College of Science, Kwame Nkrumah University of Science and Technology, Kumasi, Ghana; 2 Department of Medical Laboratory Technology, College of Health, Kintampo, Ghana; 3 Department of Medical Biochemistry and Molecular Biology, Dalian Medical University, 116044, 9 Western Section, Lvshun South Street, Lvshunkou District, Dalian City, P.R. China

**Keywords:** Metabolic syndrome, Abdominal adiposity, Insulin resistance, Postmenopausal and premenopausal women

## Abstract

**Background:**

Metabolic syndrome (MetS) is an important health problem which puts individuals at risk for cardiovascular diseases and type 2 diabetes as well as obesity-related cancers such as colon and renal cell in men, and endometrial and oesophageal in women.

**Objective:**

This study was aimed at examining how obesity indicators and related determinants influence metabolic syndrome, and how the factors can be used to predict the syndrome and its cut-offs in postmenopausal Ghanaian women.

**Methods:**

Two hundred and fifty (250) Ghanaian subjects were involved in the study with one hundred and forty-three (143) being premenopausal women and one hundred and seven (107) postmenopausal women. The influence of traditional metabolic risk factors including high blood pressure, dyslipidemia and glucose intolerance on obesity and atherogenic indices i.e. body mass index (BMI), waist circumference (WC), waist-to-hip ratio (WHR), Waist-to-thigh ratio (WTR), waist-to-height ratio (WHtR), high density lipoprotein cholesterol to total cholesterol ratio (HDL-C/TC), high density lipoprotein cholesterol to low density lipoprotein ratio (HDL-C/LDL-C) and triglyceride to high density lipoprotein cholesterol ratio (TG/HDL-C) were identified according to the Harmonization (H_MS) criterion.

**Results:**

The predominant anthropometric marker that significantly influence metabolic risk factors among the pre- and postmenopausal women was waist-to-hip ratio (premenopausal: p- 0.004, 0.026 and 0.002 for systolic blood pressure (SBP), fasting blood glucose (FBG) and HDL-C; postmenopausal: p-0.012, 0.048, 0.007 and 0.0061 for diastolic blood pressure (DBP), FBG, triglyceride (TG) and high density lipoprotein cholesterol (HDL-C) respectively). Using the receiver operating characteristic (ROC) analysis, the area under the curve for WC, WHR, TG/HDL-C and HDL-C/TC among postmenopausal women were estimated at 0.6, 0.6, 0.8 and 0.8 respectively. The appropriate cut-off values for WC, WHR, TG/HDL-C and HDL-C/TC that predicted the presence of metabolic syndrome were 80.5 cm, 0.84, 0.61 and 0.34 respectively.

**Conclusion:**

The presence of metabolic syndrome among Ghanaian postmenopausal women can be predicted using WC, WHR, TG/HDL-C and HDL-C/TC.

## Introduction

The increase in obesity, particularly abdominal adiposity, is closely associated with premature atherosclerosis and many metabolic modifications including insulin resistance, dyslipidemia hypertension and diabetes [1-3]. Obesity is the most common disorder associated with women in their menopausal stage and occurs in approximately 65% of all women [4]. The development of central obesity, insulin resistance as well as the worsening of glucose and lipid metabolism has been associated with menopause which results in an increased risk for cardiovascular disease [5,6]. According to Arad *et al.*., [7] central obesity contribute to the development of insulin resistance as well as atherosclerosis in women. Body mass index (BMI) is commonly used in public health studies as indicator of weight status but it does not consider the accumulation of abdominal visceral fats [8]. Waist circumference (WC), waist-hip ratio (WHR), waist-to-thigh ratio (WTR) and waist-height ratio (WHtR) are anthropometric measures used to diagnose abdominal obesity, while high triglyceride (TG) and low high density lipoprotein-cholesterol (HDL-C) are used to define dyslipidemia. These two markers are negatively correlated and are risk factors for atherosclerotic cardiovascular disease and stroke [9]. Since many women with the syndrome have low HDL-C, they often have low HDL-cholesterol/total cholesterol (HDL-C/TC), HDL-cholesterol/low density lipoprotein-cholesterol (HDL-C/LDL-C) and high triglycerides/HDL-cholesterol ratios [10]. These ratios had been shown to be significant predictors of atherosclerosis, both in Caucasians and Nigerian women [11]. The relationship between obesity indicators such as BMI, WC, WHR, WTR and WHtR, and traditional markers of metabolic syndrome such as fasting glucose, triglyceride, blood pressure and HDL cholesterol has not been fully established in Ghanaian population. Moreover, the association between obesity markers and atherogenic indices such as TG/HDL-C and HDL-C/TC as well as their ability to predict metabolic syndrome among Ghanaian postmenopausal women has also not been reported. This study was therefore aimed at examining how obesity indicators and related factors affect metabolic syndrome, and also to ascertain how obesity and atherogenic indices can be used to predict the syndrome and its cut-offs in Ghanaian postmenopausal women.

## Subjects, materials and methods

### Subjects

This cross-sectional study was carried out between May and July, 2011 at the outpatient departments of Suntreso and Seventh Day Adventist (SDA) Government Hospitals, Ghana. Two hundred and fifty patients were randomly recruited, of which one hundred and forty-three (143) were premenopausal women and one hundred and seven (107) postmenopausal. The study participants were recruited from a population of young and older women aged 20–78 years. Women who were still menstruating irrespective of the regularities of their menses were considered as premenopausal women while postmenopausal women were women who had ceased menstruation for at least one year. The participation of the women was voluntary. Informed consent was obtained from each of them after thorough explanation of the study in a language they understand. This study was approved (CHRPE/02/11) by the Committee on Human Research Publications and Ethics, School of Medical Sciences; Kwame Nkrumah University of Science and Technology and Komfo Anokye Teaching Hospital, Ghana

### Sample size consideration

The 250 participants were used after the estimation of minimum sample sizes for both pre (98) and postmenopausal women (88) to achieve 80% power based on the method suggested by [12,13]. The parameters used were: prevalence of metabolic syndrome among pre- and postmenopausal women 24% and 44.4% respectively [14]; 24%, confidence interval of 95%, relative sample size of 0.9, probability of type II error, 20% and probability of type I error 5%.

### Inclusion and exclusion criteria

The eligible volunteers were women with no signs of pregnancy, hypertension, type 2 diabetes, cancer, polycystic ovary syndrome, hepatitis B and hormonal contraceptive users. The exclusion criterion was any of the following: clinically-confirmed pregnancy, known diabetics, hypertension, other heart diseases, polycystic ovary syndrome and cancer.

### Laboratory procedures

Venous blood samples were collected after overnight fast (12–16 hours) between 7 am and 10 am. About 5 ml of venous blood was collected; 4 ml dispensed into vacutainer® plain tubes and 1 ml into fluoride oxalate tubes. After centrifugation at 1000 rpm for 10 minutes, the serum and plasma were stored at −80°C until assayed. Parameters determined included: FBG, TC, TG and HDL-C according to reagents manufacturer’s specification (Fortress Diagnostics Limited, Antrim, United Kingdom). Serum low density lipoprotein cholesterol and very low density lipoprotein-cholesterol (VLDL-C) were calculated using the Frederickson-Friedwald’s formula [15]. Fasting blood glucose and total cholesterol determination were according to the method described by Trinder [16]. Triglycerides determination employed a modified Trinder method. HDL-C was measured after precipitation with phosphotungstic acid in the presence of magnesium ions. Various ratios like TG/HDL-C, HDL-C/TC and LDL-C/HDL-C and HDL-C/VLDL-C were calculated using Microsoft Excel.

### Anthropometric variables

Anthropometric measurements included height to the nearest centimetre without shoes and weight to the nearest 0.1 kg in light clothing. Subjects were weighed on a bathroom scale (BR9012; Zhongshan Camry Electronic Co. Ltd, Guangdong, China) and their height measured with a wall-mounted ruler. BMI was calculated by dividing weight (kg) by height squared (m²). Waist circumference was measured at the midpoint between the last rib and the iliac crest with the participants standing and wearing light cloths with a Gulick II spring-loaded measuring tape (Gay Mills, WI). The hip circumference was measured at the widest level over the greater trochanters and the WHR calculated by dividing the waist circumference (cm) by the hip circumference (cm). Thigh circumference on the other hand was measured on the left leg below the gluteal fold and waist to thigh ratio calculated by dividing waist circumference (cm) by the thigh circumference (cm).

### Blood pressure

Blood pressure was measured by a trained nurse with participants in sitting position and having rested for at least 10 minutes using sphygmomanometer and appropriate cuff sizes. Three separate readings were taken per subject, after two minutes intervals and the lowest readings recorded. Systolic blood pressure (SBP) and diastolic blood pressure (DBP) were taken at the 1^st^ and 5^th^ Korotkoff sounds respectively. Pulse pressure was calculated using SBP-DBP.

### Definition of metabolic syndrome

#### Harmonization (H_MS)

Metabolic risk factors were identified based on the definition released by an expert group from the International Diabetes Federation (IDF), National Heart, Lung, Blood Institute (NHLBI), World Health Federation and other international associations which proposed a harmonized definition (H_MS) [17,18] that uses uniform cut-off points for all the risk factors and recommended that individuals with metabolic syndrome should have any three of the following five components: (1) waist measurement >80 cm for women; (2) TG levels of 1.7 mmol/ L or greater, (3) HDL-C lower than 1.29 mmol/ L for women, (4) BP of 130/85 mm Hg or greater and (5) FBG of 5.6 mmol/L or greater. The pre- and postmenopausal women were categorized using this criterion.

### Statistical analyses

Normality of all variables was tested and found to be normal before the statistical analyses using the D’ Agostino-Pearson procedure. All clinical and biochemical data of study subjects were expressed as means ± SEM. The differences between groups were examined by unpaired t-test. Areas under the curve (AUC) for the atherogenic and obesity markers were measured through ROC curve analysis for the diagnosis of metabolic syndrome amongst pre- and postmenopausal women. The diagnostic performance characteristics in terms of sensitivity and specificity were calculated at different cut-offs for those markers which showed higher AUC. The significance of the difference between the area under the curves derived from pre and postmenopausal samples were calculated using the formula suggested by [19]. To compare differences between premenopausal and postmenopausal women with and without the syndrome, one way analysis of variance (ANOVA) followed by Tukey’s multiple test to compare all pairs of columns were performed. All p values were two-sided and the level of significance was 0.05 after Bonferroni correction [20]. GraphPad Prism version 5.00 (GraphPad software, San Diego California, USA; http://www.graphpad.com) and Statistical Package for the Social Sciences (SPSS) version 16.00 for windows (SPSS Inc, Chicago, USA; http://www.spss.com) were used for statistical analysis.

## Results

### Baseline characteristics of the study population

Table 1 shows the baseline characteristics of the study population. The mean age of postmenopausal (57.25 ± 0.8) was significantly higher (*p* < 0.0001) than the mean age of the premenopausal (34.48 ± 0.7). Postmenopausal women had significantly (p < 0.0001) larger WC, higher mean WHR, WTR, WHtR, SBP, DBP and PP than their premenopausal counterpart except BMI (p = 0.4152, Table 1). Compared to premenopausal women, postmenopausal counterparts had significantly (p < 0.05) raised levels of TG, VLDL-C, and FBG, though these levels were within normal range. The mean TG/HDL-C ratio was higher (p = 0.0040) among the postmenopausal group (Table 1). However, postmenopausal women had reduced HDL-C as compared to their premenopausal counterparts though not statistically significant (Table 1).

**Table 1:** Baseline characteristics of study population

**Characteristics**	**Total**	**Postmenopausal**	**Premenopausal**	** *P* value**
** *Number of Subjects* **	250	107	143	
Age (years)	44.23 ± 0.90	57.25 ± 0.80	34.48 ± 0.74	<0.0001
Waist Circ. (cm)	92.41 ± 0.72	95.93 ± 0.94	89.85 ± 1.01	<0.0001
Thigh Circ. (cm)	55.64 ± 0.41	56.43 ± 0.80	55.34 ± 0.53	0.2380
BMI (kg/m²)	26.64 ± 0.32	27.25 ± 0.57	26.41 ± 0.44	0.2350
WHR	0.88 ± 0.00	0.91 ± 0.01	0.87 ± 0.01	<0.0001
WTR	1.67 ± 0.01	1.72 ± 0.01	1.62 ± 0.01	<0.0001
HTR	1.88 ± 0.01	1.95 ± 0.06	1.87 ± 0.01	0.1836
WHtR	0.58 ± 0.00	0.60 ± 0.01	0.56 ± 0.01	<0.0001
SBP (mmHg)	132.5 ± 1.19	140.1 ± 1.81	126.4 ± 1.44	<0.0001
DBP (mmHg)	86.3 ± 0.73	89.59 ± 1.12	83.62 ± 0.93	<0.0001
PP (mmHg)	46.2 ± 0.76	50.76 ± 1.12	42.73 ± 0.93	<0.0001
FBG (mmol/l)	5.19 ± 0.08	5.60 ± 0.15	4.9 ± 0.07	<0.0001
TG (mmol/l)	1.20 ± 0.03	1.31 ± 0.06	1.12 ± 0.04	0.0060
TC (mmol/l)	4.40 ± 0.05	4.41 ± 0.08	4.40 ± 0.07	0.8500
HDL-C (mmol/l)	1.34 ± 0.02	1.31 ± 0.03	1.37 ± 0.02	0.0670
LDL-C (mmol/l)	2.51 ± 0.05	2.50 ± 0.08	2.52 ± 0.06	0.9130
VLDL-C (mmol/l)	0.42 ± 0.01	0.46 ± 0.02	0.40 ± 0.01	0.0060
HDL-C/TC	0.31 ± 0.00	0.30 ± 0.01	0.32 ± 0.01	0.0700
HDL-C/LDL-C	0.59 ± 0.02	0.84 ± 0.13	0.64 ± 0.04	0.0970
TG/HDL-C	0.97 ± 0.04	1.11 ± 0.08	0.86 ±0.04	0.0040

Continuous data were presented as mean ± standard error of mean (SEM). *VLDL-C*: Very Low Density Lipoprotein-Cholesterol, *LDL-C*: Low Density Lipoprotein-Cholesterol, *HDL-C*: High Density Lipoprotein, *TC*: Total Cholesterol, *TG*: Triglycerides, *FBG*: Fasting Blood Glucose, *WHR*: Waist-to-Hip Ratio, *BMI*: Body Mass Index, *WC*: Waist Circumference, *WTR*: Waist-to-Thigh Ratio, *WHtR*: Waist-to-Height Ratio, *HTR*: hip-to-thigh circumference, *SBP*: Systolic Blood Pressure, *DBP*: Diastolic Blood Pressure, *PP*: Pulse Pressure, *Circ*: Circumference.

### Influence of obesity markers on metabolic risk factors

Table 2 shows the influence of BMI, WC, WTR, WHR, WHtR on the cut-offs of BP, FBG, TG, and HDL-C in postmenopausal and premenopausal women separately using H_MS criterion. Postmenopausal women with elevated BMI and WHR (p = 0.0010 and 0.0120 respectively) had raised SBP (>130 mmHg), whereas premenopausal women with high BMI, WC, WHR, WTR and WHtR (p = 0.0030, 0.0004, 0.0004, 0.0030 and 0.0010 respectively) had raised SBP (>130 mmHg) respectively (Table 2). Postmenopausal women with high WC, WHR, and WHtR had significantly (p = 0.0060, 0.0480, <0.0001 and 0.0003 respectively) raised FBG levels (≥5.6 mmol/l) while high WTR (p < 0.0001) was rather linked to low FBG (less than 5.6 mmol/l). On the other hand, among premenopausal subjects, only elevated WHR (p = 0.0260) was associated with raised fasting blood glucose (Table 2). Similarly, elevated WC, WHR, less WTR and WHtR (p = 0.0040, 0.0070, 0.0320 and 0.0070 respectively) were linked to raise triglyceride (≥1.7 mmol/l) among postmenopausal women. Finally, only WHR (p = 0.0060 and 0.0020 respectively) had influence on reduced HDL-C (<1.30 mmol/l) among postmenopausal and premenopausal populations (Table 2).

**Table 2:** BMI, WC, WTR, WHR and WHtR values according to the various cut-offs of different metabolic risk factors in pre- and postmenopausal Ghanaian women using H_MS Criterion

	**Postmenopausal**					**Premenopausal**				
**Parameters**	**BMI**	**WC**	**WHR**	**WTR**	**WHtR**	**BMI**	**WC**	**WHR**	**WTR**	**WHtR**
Systolic Blood Pressure										
<130 mmHg	24.19 ± 0.80	92.50 ± 1.80	0.92 ± 0.01	1.70 ± 0.02	0.58 ± 0.01	25.37 ± 0.50	87.10 ± 1.10	0.85 ± 0.01	1.60 ± 0.01	0.54 ± 0.01
≥130 mmHg	27.65 ± 0.50	96.80 ± 1.10	0.91 ± 0.01	1.73 ± 0.02	0.61 ± 0.01	28.11 ± 0.80	94.39 ± 1.90	0.89 ± 0.01	1.67 ± 0.02	0.59 ± 0.01
** *p value* **	0.0010	0.0530	0.5830	0.2540	0.1360	0.0030	0.0004	0.0004	0.0030	0.0010
Diastolic Blood Pressure										
<85 mmHg	24.59 ± 0.70	94.00 ± 1.60	0.93 ± 0.01	1.70 ± 0.02	0.59 ± 0.01	25.10 ± 0.50	86.96 ± 1.10	0.86 ± 0.01	1.62 ± 0.01	0.54 ± 0.01
≥85 mmHg	27.86 ± 0.60	96.61 ± 1.10	0.91 ± 0.01	1.73 ± 0.02	0.60 ± 0.01	28.34 ± 0.80	93.97 ± 1.80	0.87 ± 0.01	1.65 ± 0.02	0.59 ± 0.01
** *p value* **	0.0010	0.1990	0.0120	0.3430	0.7580	0.00020	0.0010	0.1180	0.2040	0.0010
Fasting Blood Glucose										
<5.6 mmol/l	26.64 ± 0.60	93.91 ± 1.20	0.91 ± 0.01	1.76 ± 0.02	0.58 ± 0.01	26.81 ± 0.50	90.04 ± 1.10	0.86 ± 0.01	1.62 ± 0.01	0.56 ± 0.01
≥5.6 mmol/l	27.13 ± 0.70	99.18 ± 1.50	0.93 ± 0.01	1.66 ± 0.01	0.63 ± 0.01	24.98 ± 0.90	88.87 ± 2.30	0.89 ± 0.01	1.63 ± 0.02	0.55 ± 0.02
** *p value* **	0.6060	0.0060	0.0480	<0.0001	0.0003	0.1400	0.6720	0.0260	0.7090	0.7290
Triglyceride										
<1.7 mmol/l	26.62 ± 0.50	94.76 ± 1.00	0.91 ± 0.01	1.73 ± 0.01	0.59 ± 0.01	26.30 ± 0.50	89.37 ± 1.04	0.87 ± 0.01	1.62 ± 0.01	0.56 ± 0.01
≥1.7 mmol/l	28.32 ± 1.00	102.4 ± 1.50	0.95 ± 0.01	1.65 ± 0.02	0.64 ± 0.01	28.21 ± 1.70	97.00 ± 3.60	0.89 ± 0.02	1.64 ± 0.01	0.60 ± 0.03
** *p value* **	0.1930	0.0040	0.0070	0.0320	0.0070	0.2970	0.0670	0.1540	0.7600	0.0890
HDL-C										
<1.30 mmol/l	26.41 ± 0.60	96.76 ± 1.40	0.93 ± 0.01	1.69 ± 0.02	0.61 ± 0.01	26.09 ± 0.80	90.97 ± 2.00	0.89 ± 0.01	1.62 ± 0.01	0.57 ± 0.01
≥1.30 mmol/l	26.98 ± 0.60	95.34 ± 1.20	0.90 ± 0.01	1.74 ± 0.02	0.59 ± 0.01	26.56 ± 0.50	89.49 ± 1.20	0.86 ± 0.01	1.62 ± 0.01	0.56 ± 0.01
** *p value* **	0.5530	0.4710	0.00610	0.0690	0.3450	0.6510	0.5310	0.0020	0.9820	0.4920

Continuous data were presented as mean ± standard error of mean (SEM). *HDL-C*: High Density Lipoprotein, *WHR*: Waist-to-Hip Ratio, *BMI*: Body Mass Index, *WC*: Waist Circumference, *WTR*: Waist-to-Thigh Ratio, *WHtR*: Waist-to-Height Ratio.

### Influence of atherogenic indices on metabolic risk factors

The influence of atherogenic indices such as TG/HDL-C, HDL-C/TC and HDL-C/LDLC and traditional metabolic risk factors is shown in Table 3. Premenopausal women (p < 0.0001 and 0.0140 respectively) with raised blood pressure (>130 mmHg) had increased TG/HDL-C and HDL-C/LDL-C ratios (Table 3). Among postmenopausal women, raised fasting blood glucose and triglyceride levels were common with higher TG/HDL-C and HDL-C/LDL-C ratios (p < 0.0001, 0.0470, <0.0001 and 0.0010 respectively) but only with TG/HDL-C ratio (p < 0.0001 for both) in premenopausal groups (Table 3). Finally, reduced levels of HDL-C were noticeable in elevated TG/HDL-C ratio (p < 0.0001) and reduced HDL-C/TC ratio (p < 0.0001) in postmenopausal group whereas it was apparent in higher TG/HDL-C ratio (p < 0.0001) but in lower HDL-C/TC ratios (p < 0.0001 and 0.0010 respectively) among premenopausal subjects (Table 3).

**Table 3:** TG/HDL-C, HDL-C/TC and HDL-C/LDL-C values according to the various cut-offs of different metabolic risk factors in pre- and postmenopausal Ghanaian women using H_MS Criterion

		**Postmenopausal**			**Premenopausal**	
**Parameters**	**TG/HDL-C**	**HDL-C/TC**	**HDL-C/LDL-C**	**TG/HDL-C**	**HDL-C/TC**	**HDL-C/LDL-C**
Systolic Blood Pressure						
<130 mmHg	1.06 ± 0.11	0.33 ± 0.02	1.17 ± 0.48	0.71 ± 0.02	0.31 ± 0.01	0.57 ± 0.03
≥130 mmHg	1.11 ± 0.10	0.31 ± 0.01	0.74 ± 0.09	1.13 ± 0.09	0.34 ± 0.01	0.76 ± 0.08
** *p value* **	0.8290	0.5800	0.1490	<0.0001	0.0980	0.0140
Diastolic Blood Pressure						
<85 mmHg	1.12 ± 0.10	0.33 ± 0.02	1.14 ± 0.37	0.71 ± 0.02	0.32 ± 0.01	0.58 ± 0.03
≥85 mmHg	1.09 ± 0.11	0.31 ± 0.01	0.70 ± 0.09	1.09 ± 0.08	0.33 ± 0.01	0.77 ± 0.08
** *p value* **	0.8640	0.2410	0.1070	<0.0001	0.2290	0.0210
Fasting Blood Glucose						
<5.6 mmol/l	0.72 ± 0.02	0.33 ± 0.01	0.64 ± 0.05	0.75 ± 0.02	0.32 ± 0.01	0.63 ± 0.04
≥5.6 mmol/l	1.76 ± 0.18	0.29 ± 0.02	1.16 ± 0.33	1.47 ± 0.18	0.33 ± 0.02	0.66 ± 0.09
** *p value* **	<0.0001	0.0880	0.0470	<0.0001	0.8380	0.7730
Triglyceride						
<1.7 mmol/l	0.84 ± 0.03	0.32 ± 0.01	0.66 ± 0.05	0.77 ± 0.02	0.33 ± 0.01	0.65 ± 0.04
≥1.7 mmol/l	2.69 ± 0.32	0.28 ± 0.03	1.87 ± 0.81	2.26 ± 0.27	0.28 ± 0.03	0.56 ± 0.12
** *p value* **	<0.0001	0.1850	0.0010	<0.0001	0.1040	0.5550
HDL-C						
<1.30 mmol/l	1.74 ± 0.20	0.24 ± 0.01	0.65 ± 0.17	1.26 ± 0.14	0.25 ± 0.01	0.44 ± 0.04
≥1.30 mmol/l	0.77 ± 0.03	0.36 ± 0.01	0.93 ± 0.17	0.74 ± 0.02	0.35 ± 0.01	0.72 ± 0.05
** *p value* **	<0.00010	<0.00010	0.2990	<0.00010	<0.00010	0.0010

Continuous data were presented as mean ± standard error of mean (SEM). *LDL-C*: Low Density Lipoprotein-Cholesterol, *HDL-C*: High Density Lipoprotein, *TC*: Total Cholesterol, *TG*: Triglycerides.

### Comparison of metabolic indicators among pre- and postmenopausal women with and without metabolic syndrome

Table 4 presents the comparison of metabolic indicators among pre- and postmenopausal women with and without (PRWM, PRWtM, POWM and POWtM) the syndrome. Systolic blood pressure was significantly higher among both postmenopausal group and PRWM than PRWtM. Similarly, fasting blood glucose levels of POWM and PRWM were significantly higher than POWtM and PRWtM (Table 4). Postmenopausal women with the syndrome had significantly raised triglyceride levels as compare to all the other groups. However, HDL-C levels were significantly reduced among POWM and PRWM than their counterparts without the syndrome. The ratios of HDL-C/VLDL-C and HDL-C/TC were significantly lower in Ghanaian women with the syndrome than those without the syndrome. Finally, the values of WHR, WTR and WHtR were significantly higher in postmenopausal groups than those in premenopausal groups (Table 4).

**Table 4:** Comparison of Metabolic Indicators among Pre and Postmenopausal Women with and without Metabolic Syndrome

**Parameters**	**POWtM**	**POWM**	**PRWM**	**PRWtM**	**p value**
	**(n = 60)**	**(n = 47)**	**(n = 29)**	**(n = 114)**	
** *H_MS* **					
Age (yrs)	57.53 ± 1.13^‡‡‡***^	56.89 ± 1.13^###§§§^	38.38 ± 1.64^¶^	33.49 ± 0.81	<0.0001
SBP (mmHg)	142.0 ± 2.56^***^	138.9 ± 2.26^§§§^	134.0 ± 4.02^¶^	124.4 ± 1.44	<0.0001
DBP (mmHg)	91.33 ± 1.64	88.00 ± 1.28^###^	85.48 ± 2.48	83.19 ± 0.98	<0.0001
FBG (mmol/l)	4.78 ± 0.11^‡‡‡^	6.59 ± 0.23^†††§§§^	6.03 ± 0.21^¶¶¶^	4.61 ± 0.05	<0.0001
TG (mmol/l)	1.10 ± 0.02^‡^	1.57 ± 0.13^†††§§§^	1.46 ± 0.15^¶¶^	1.05 ± 0.02	<0.0001
HDL-C (mmol/l)	1.49 ± 0.02^†††‡‡‡^	1.08 ± 0.04	1.12 ± 0.04	1.43 ± 0.02^§§§¶¶¶^	<0.0001
HDL-C:TC ratio	0.33 ± 0.01^†††^	0.27 ± 0.01	0.29 ± 0.02	0.32 ± 0.01^§§§^	<0.0001
TG:HDL-C ratio	0.74 ± 0.01	1.58 ± 0.16^†††§§§^	1.34 ± 0.15^‡‡‡¶¶¶^	0.74 ± 0.02	<0.0001
HDL-C:LDL-C ratio	0.59 ± 0.01	0.57 ± 0.05	0.65 ± 0.09	0.58 ± 0.01	0.5519
HDL-C:VLDL-C ratio	3.89 ± 0.08^†††‡‡‡^	2.59 ± 0.22	2.82 ± 0.29	4.03 ± 0.09^§§§¶¶¶^	<0.0001
BMI (kg/m²)	26.52 ± 0.64	27.44 ± 0.64	26.40 ± 0.77	26.48 ± 0.52	0.7041
WHR	0.90 ± 0.01^***^	0.93 ± 0.01^##§§§^	0.89 ± 0.01	0.86 ± 0.01	<0.0001
WTR	1.75 ± 0.02^‡‡‡***^	1.68 ± 0.02	1.63 ± 0.02	1.62 ± 0.01	<0.0001
WHtR	0.59 ± 0.01^*^	0.61 ± 0.01^##§§§^	0.56 ± 0.01	0.55 ± 0.01	<0.0001

Data were presented as mean ± standard error of mean (SEM). *POWtM*: postmenopausal women without the syndrome, *POWM*: postmenopausal women with the syndrome, *PRWM*: premenopausal women with the syndrome, *PRWtM*: premenopausal women without the syndrome, *LDL-C*: Low Density Lipoprotein-Cholesterol, *HDL-C*: High Density Lipoprotein, *TC*: Total Cholesterol, *TG*: Triglycerides, *FBG*: Fasting Blood Glucose, *WHR*: Waist-to-Hip Ratio, *BMI*: Body Mass Index, *WTR*: Waist-to-Thigh Ratio, *WHtR*: Waist-to-Height Ratio, *SBP*: Systolic Blood Pressure, *DBP*: Diastolic Blood Pressure, *WHR*: Waist-to-Hip Ratio, *BMI*: Body Mass Index, *WC*: Waist Circumference, *THC*: Thigh Circumference, *WTR*: Waist-to-Thigh Ratio, *WHtR*: Waist-to-Height Ratio, *HTR*: hip-to-thigh circumference. *Each comparison was performed between pre and postmenopausal women with and without metabolic syndrome. .*p < 0.05, **p < 0.001, ***p < 0.0001, comparison of POWtM with PRWtM; †p < 0.05, †† p < 0.001, ††† p < 0.0001, comparison of POWtM with POWM; ‡ p < 0.05, ‡‡ p < 0.001, ‡‡‡ p < 0.0001, comparison of POWtM with PRWM; ¶ p < 0.05, ¶¶ p < 0.001, ¶¶¶ p < 0.0001, comparison of PRWM with PRWtM; § p < 0.05, §§ p < 0.001, §§§ p < 0.0001, comparison of POWM with PRWtM; # p < 0.05, ## p < 0.001, ### p < 0.0001, comparison of POWM with PRWM.

### Prediction of metabolic syndrome among pre- and postmenopausal women using atherogenic and obesity indices

**Figure 1:**
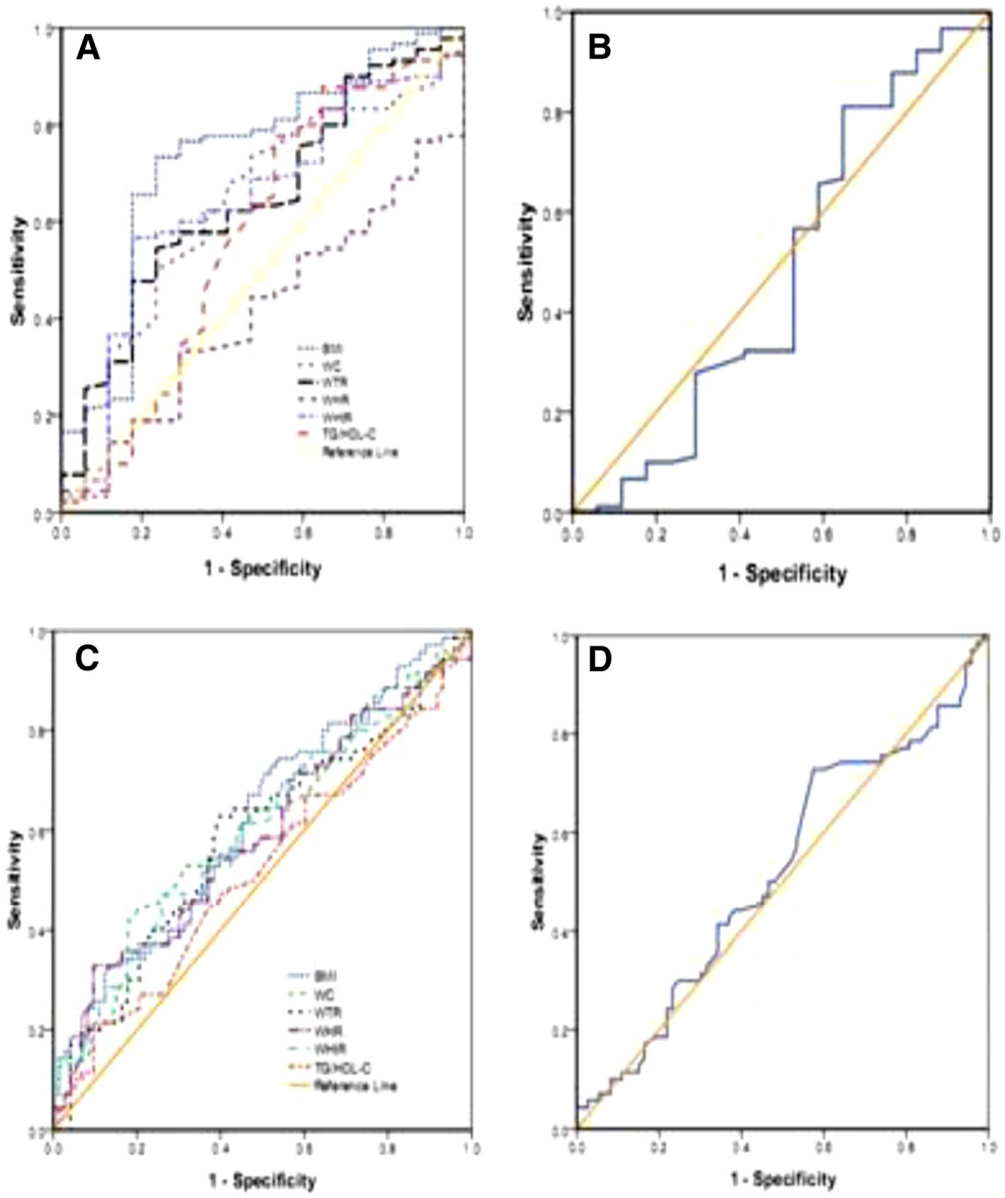
**The ROC (receiver operating characteristic) curves A, B, C, and D for BMI, WC, WHtR, WTR, WHR, and TG/HDL-C as well as HDL-C/TC and TG/HDL-C to detect high blood pressure in postmenopausal and premenopausal Ghanaian Women respectively**.

**Figure 2:**
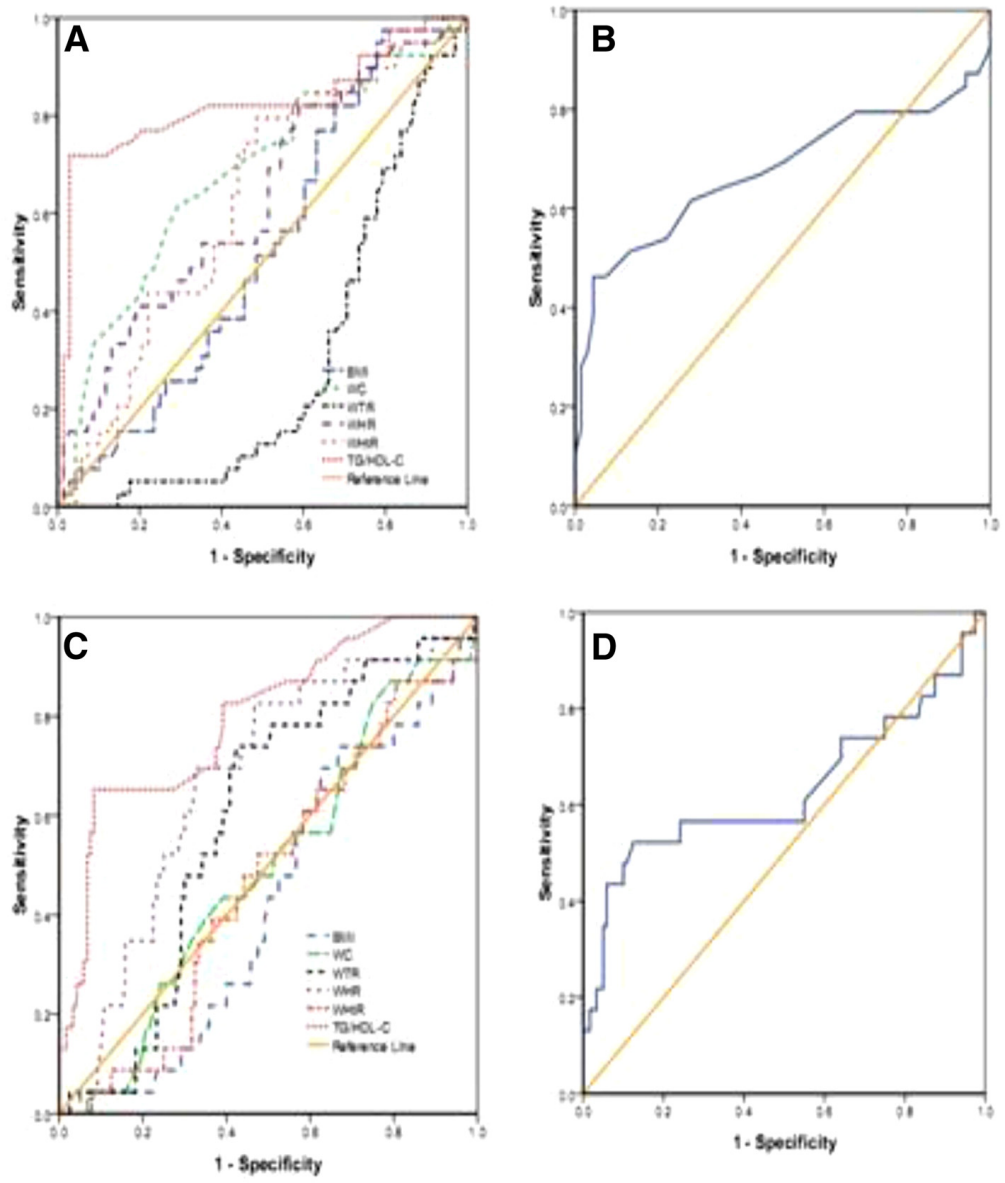
**The ROC (receiver operating characteristic) curves A, B, C, and D for BMI, WC, WHtR, WTR, WHR, and TG/HDL-C as well as HDL-C/TC and TG/HDL-C to detect high glucose level in postmenopausal and premenopausal Ghanaian Women respectively**.

**Figure 3:**
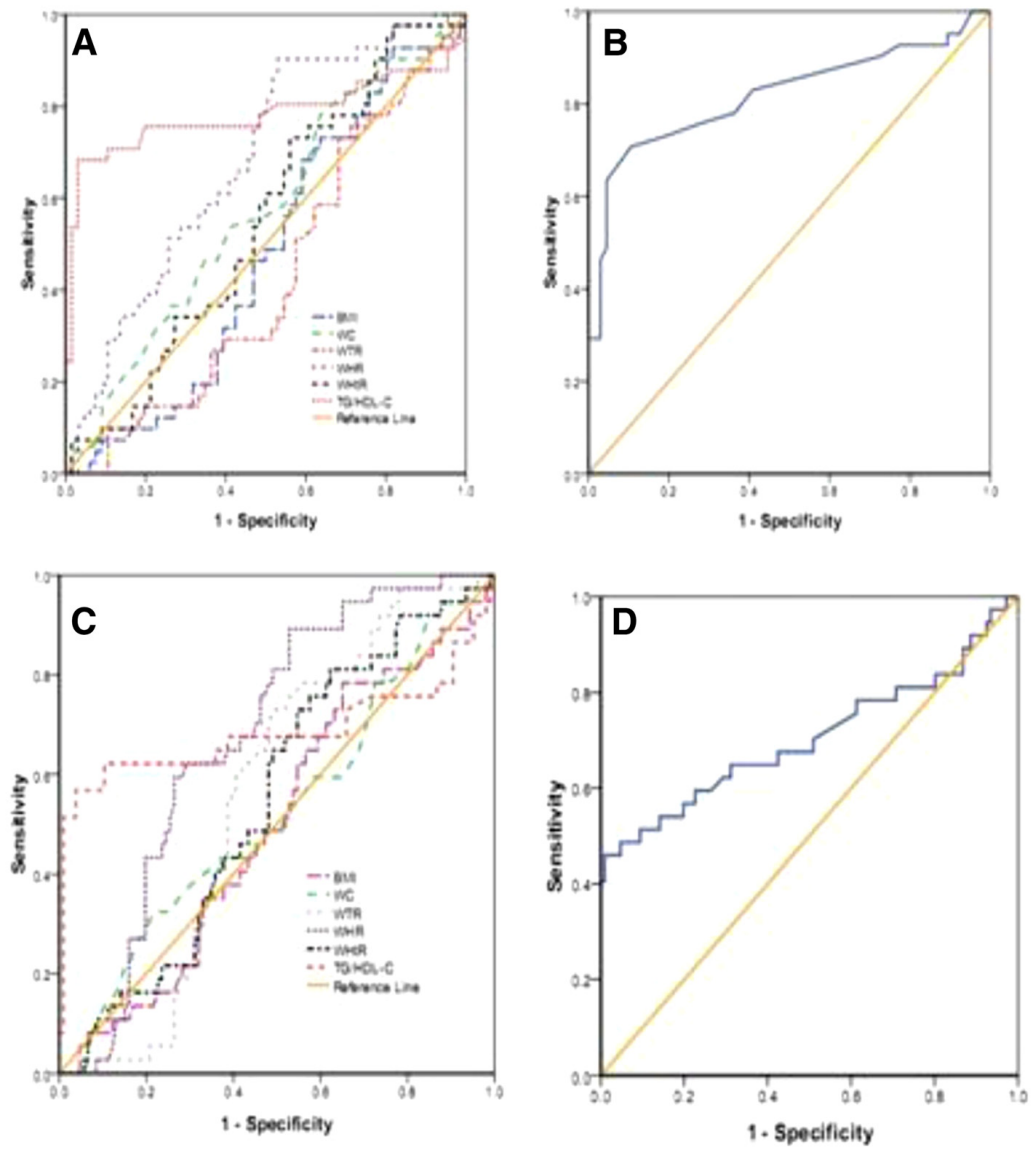
**The ROC (receiver operating characteristic) curves A, B, C, and D for BMI, WC, WHtR, WTR, WHR, and TG/HDL-C as well as HDL-C/TC and TG/HDL-C to detect low HDL-C level in postmenopausal and premenopausal Ghanaian Women respectively**.

**Figure 4:**
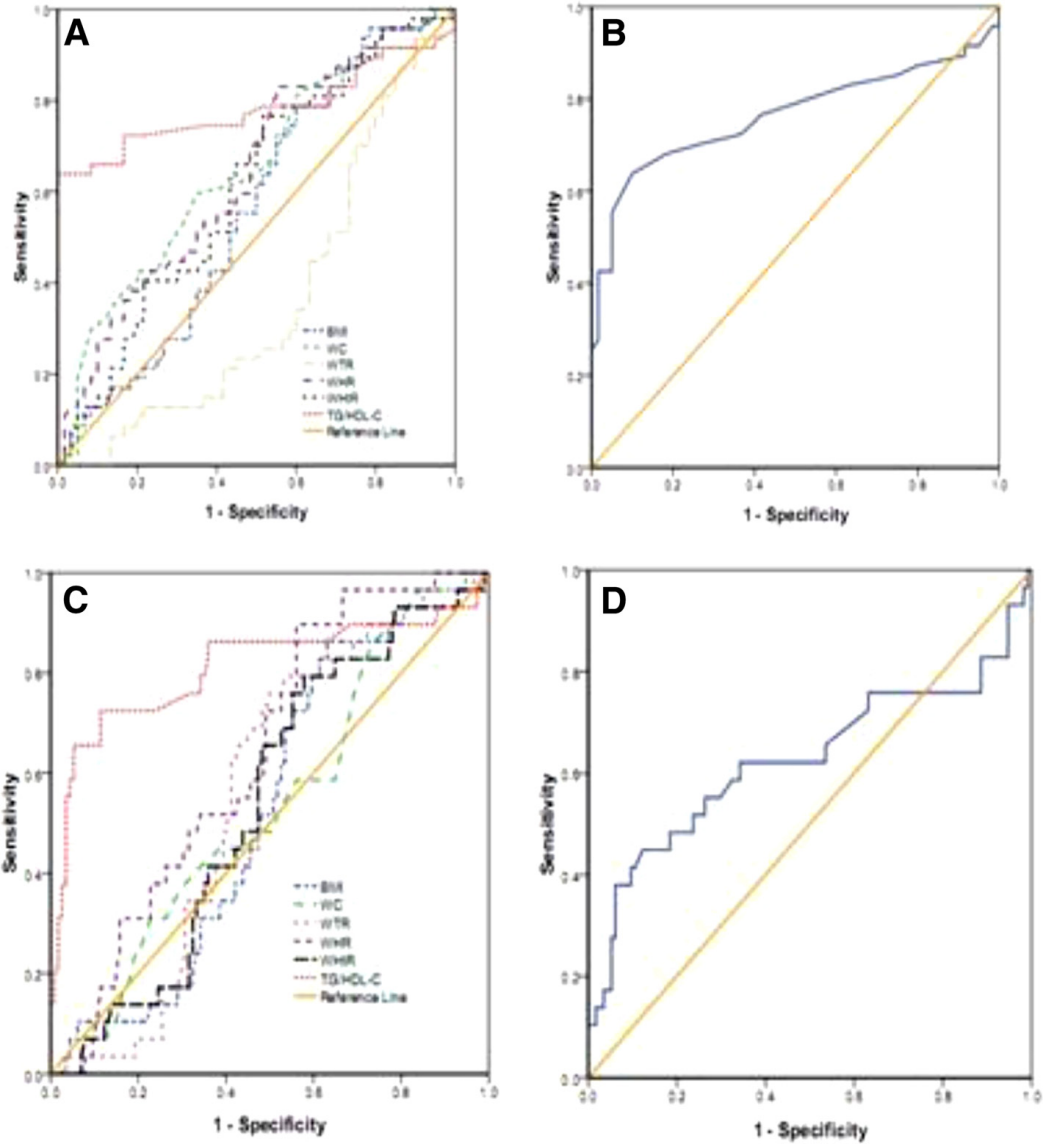
**The ROC (receiver operating characteristic) curves A, B, C, and D for BMI, WC, WHtR, WTR, WHR, and TG/HDL-C as well as HDL-C/TC and TG/HDL-C to detect metabolic syndrome among postmenopausal and premenopausal Ghanaian Women respectively**.

The area under curves (AUCs) of those lipid and obesity-based markers which showed significant prediction of high blood pressure, fasting blood glucose, low HDL-C and metabolic syndrome is shown in Tables 5 and Figures 1, 2, 3 and 4.

**Table 5:** Area under Curves of BMI, WC, WTR, WHR, TG/HDL-C, HDL-C/TC and WHtR for Metabolic Syndrome

	**BMI**	**WC**	**WTR**	**WHR**	**WHtR**	**TG/HDL-C**	**HDL-C/TC**
** *POSTMENOPAUSAL* **							
Blood Pressure	0.7(0.6-0.9)*	0.6(0.5-0.8)	0.7(0.5-0.8)*	0.4(0.3-0.6)	0.7(0.5-0.8)*	0.6(0.4-0.7)	0.5(0.4-0.7)
Glucose	0.5(0.4-0.6)	0.7(0.6-0.8)*	0.3(0.2-0.4)	0.6(0.5-0.7)*	0.6(0.5-0.7)*	0.8(0.7-0.9)***	0.7(0.6-0.8)*
HDL-C	0.5(0.4-0.6)	0.6(0.5-0.7)	0.4(0.3-0.5)	0.7(0.6-0.8)*	0.5(0.4-0.6)	0.8(0.7-0.9)***	0.8(0.7-0.9)***
Metabolic Syndrome	0.6(0.5-0.7)	0.6(0.5-0.7)*	0.4(0.3-0.5)	0.6(0.5-0.7)*	0.6(0.5-0.7)	0.8(0.7-0.9)***	0.8(0.7-0.9)***
** *PREMENOPAUSAL* **							
Blood Pressure	0.6(0.5-0.7)*	0.6(0.5-0.7)*	0.6(0.5-0.7)	0.6(0.5-0.7)*	0.6(0.5-0.7)*	0.5(0.4-0.6)	0.5(0.4-0.6)
Glucose	0.4(0.3-0.5)	0.5(0.4-0.6)	0.6(0.5-0.7)	0.7(0.6-0.8)*	0.5(0.4-0.6)	0.8(0.7-0.9)***	0.6(0.5-0.8)*
HDL-C	0.5(0.4-0.6)	0.5(0.4-0.6)	0.6(0.5-0.7)	0.7(0.6-0.8)*	0.5(0.4-0.6)	0.7(0.6-0.8)*	0.7(0.6-0.8)***
Metabolic Syndrome	0.5(0.4-0.6)	0.5(0.4-0.6)	0.6(0.5-0.7)	0.6(0.5-0.7)*	0.5(0.4-0.6)	0.8(0.7-0.9)***	0.6(0.5-0.8)*

All values were AUC (95%CI), AUC, area under curves; CI, confidence interval, *P < 0.05. **P < 0.001 **P < 0.0001 Continuous data were presented as mean ± standard error of mean (SEM). *HDL-C*: High Density Lipoprotein, *WHR*: Waist-to-Hip Ratio, *BMI*: Body Mass Index, *WTR*: Waist-to-Thigh Ratio, *WHtR*: Waist-to-Height Ratio, *WHR*: Waist-to-Hip Ratio, *BMI*: Body Mass Index, *WC*: Waist Circumference, *WTR*: Waist-to-Thigh Ratio, *WHtR*: Waist-to-Height Ratio. Each comparison was performed between pre and postmenopausal women with and without metabolic syndrome.

In postmenopausal subjects with the cut-off value of 23.1 kg/m² (for BMI), 1.58 (for WTR), 0.53 (for WHtR), the sensitivity and specificity were 81.8% and 57.9%, 93.2% and 84.2%, 88.6% and 73.7%, respectively, which were found to detect high blood pressure (Table 6). The cutoff values for detecting fasting blood glucose were 81.5 cm (for WC), 0.84 (for WHR), 0.51 (for WHtR), 0.60 (for TG/HDL-C), 0.34 (for HDL-C/TC) and the corresponding sensitivity and specificity were 94.9% and 89.7%, 97.4% and 97.1%, 97.4% and 89.7%, 97.4% and 89.7%, 79.5% and 67.6% respectively in postmenopausal women (Table 7). The cut-off values to detect low HDL-C were 0.85 for WHR (sensitivity and specificity were 97.4% and 89.9%), 0.63 for TG/HDL-C (sensitivity and specificity were 89.5% and 87%), 0.32 for HDL-C/TC (sensitivity and specificity were 81.6% and 36.2%) (Table 8). The cut-off values to detect metabolic syndrome in postmenopausal women were 80.5 cm (for WC), 0.84 (for WHR), 0.61 (for TG/HDL-C), and the corresponding sensitivity and specificity were 95.7% and 91.7%, 97.9% and 93.3%, 87.2% and 80%, 91.5% and 88.3%, respectively (Table 9).

**Table 6:** Cutoffs of the Obesity indicators to predict High Blood Pressure in Pre- and Postmenopausal Ghanaian

**Parameters**	**Cut-off**	**Sensitivity**	**Specificity**
** *POSTMENOPAUSAL* **			
BMI (Kg/m²)	23.1	0.818	0.579
WTR	1.58	0.932	0.842
WHtR	0.53	0.886	0.737
** *PREMENOPAUSAL* **			
BMI (Kg/m²)	22.9	0.817	0.681
WC (cm)	80.5	0.803	0.722
WHR	0.80	0.915	0.861
WHtR	0.50	0.817	0.778

**Table 7:** Cutoffs of the Atherogenic and Obesity indicators to predict High Glucose Level in Pre- and Postmenopausal Ghanaian

**Parameters**	**Cut-off**	**Sensitivity**	**Specificity**
** *POSTMENOPAUSAL* **			
WC (cm)	81.5	0.949	0.897
WHR	0.84	0.974	0.971
WHtR	0.51	0.974	0.897
TG/HDL-C	0.60	0.974	0.897
HDL-C/TC	0.34	0.795	0.676
** *PREMENOPAUSAL* **			
WHR	0.82	0.913	0.783
TG/HDL-C	0.65	0.957	0.708
HDL-C/TC	0.33	0.565	0.467

**Table 8:** Cutoffs of the Atherogenic and Obesity indicators to predict Low HDL-C in Pre- and Postmenopausal Ghanaian

**Parameters**	**Cut-off**	**Sensitivity**	**Specificity**
** *POSTMENOPAUSAL* **			
WHR	0.85	0.974	0.899
TG/HDL-C	0.63	0.895	0.870
HDL-C/TC	0.32	0.816	0.362
** *PREMENOPAUSAL* **			
WHR	0.80	0.973	0.858
TG/HDL-C	0.58	0.865	0.906
HDL-C/TC	0.34	0.757	0.613

**Table 9:** Cutoffs of the Atherogenic and Obesity indicators to predict MetS in Pre- and Postmenopausal Ghanaian

**Parameters**	**Cut-off**	**Sensitivity**	**Specificity**
** *POSTMENOPAUSAL* **			
WC	80.5 cm	0.957	0.917
WHR	0.84	0.979	0.933
TG/HDL-C	0.61	0.872	0.800
HDL-C/TC	0.34	0.915	0.883
** *PREMENOPAUSAL* **	0.81	0.966	0.833
WHR			
TG/HDL-C	0.58	0.931	0.886
HDL-C/TC	0.34	0.724	0.632

Based upon the studied data, the various cut-offs with their sensitivities and specificities for premenopausal women are presented in Tables 6, 7, 8 and 9 whereas Tables 10 and 11 show the comparison of area under ROC curves between pre- and postmenopausal women.

**Table 10:** The comparison of ROC curves for blood pressure and fasting blood glucose between premenopausal and postmenopausal women

**Variables**	**Premenopausal**	**Postmenopausal**	**p value**
** *Blood Pressure* **			
BMI	0.621	0.733	0.1339
WC	0.608	0.627	0.8225
WTR	0.587	0.646	0.4775
WHR	0.589	0.418	0.0617
WHTR	0.596	0.641	0.5873
TG/HDL-C	0.521	0.571	0.5691
HDL-C/TC	0.520	0.476	0.6299
** *Fasting Blood Glucose* **			
BMI	0.427	0.524	0.2585
WC	0.492	0.680	0.0286
WTR	0.593	0.290	0.0003
WHR	0.669	0.629	0.6467
WHTR	0.468	0.623	0.0733
TG/HDL-C	0.796	0.824	0.7032
HDL-C/TC	0.631	0.674	0.6209

All values were area under the curve (AUC).

**Table 11:** The comparison of ROC curves for high density lipoprotein cholesterol and metabolic syndrome between premenopausal and postmenopausal women

**Variables**	**Premenopausal**	**Postmenopausal**	**p value**
** *High Density Lipoprotein Cholesterol* **			
BMI	0.499	0.474	0.7534
WC	0.523	0.569	0.5653
WTR	0.56	0.427	0.0932
WHR	0.675	0.681	0.9376
WHTR	0.549	0.547	0.9801
TG/HDL-C	0.685	0.782	0.1777
HDL-C/TC	0.704	0.818	0.0995
** *Metabolic Syndrome* **			
BMI	0.527	0.562	0.6725
WC	0.533	0.646	0.1655
WTR	0.563	0.364	0.0142
WHR	0.634	0.642	0.9217
WHTR	0.54	0.61	0.395
TG/HDL-C	0.81	0.782	0.684
HDL-C/TC	0.625	0.764	0.0716

All values were area under the curve (AUC).

## Discussion

Obesity and insulin resistance have been suggested to play important pathophysiological role in the etiology of metabolic syndrome as well as diseases connected to it [21,22]. The accumulation of fat in intra-abdominal depot is more common in postmenopausal women than their premenopausal counterparts and hence postmenopausal subjects have a greater risk of developing metabolic complications such as type 2 diabetes, hypertension, atherosclerosis and coronary artery disease (CAD) as well as obesity-related cancers [23]. Central obesity progressively increases hepatic and adipose-tissue insulin resistance and its resultant metabolic abnormalities like glucose intolerance, low HDL-C, elevated TG and hypertension [24,25]. Two hypotheses have been proposed in several studies [26-28] to explain the strong relationship between intra-abdominal fat accumulation and insulin resistance. Foremost, intra-abdominal adiposities are more biologically active and are located near portal vein which carries blood from the intestinal area to the liver. Substances released by intra-abdominal fat, including free fatty acids enter the portal circulation and to the liver and subsequently influence glucose metabolism as well as blood lipids production [29]. Secondly, visceral adipose tissue and its resident macrophages produce more inflammatory cytokines like tumor necrosis factor-alpha (TNF- α) and interleukin-6 (IL-6) and less adiponectin [30]. The change in levels of cytokines induces insulin resistance by depressing the synthesis of glucose transport protein, GLUT 4.

This present study suggests that WC, WHR, TG/HDL-C as well as HDL-C/TC values are significant indicators to identify the presence of metabolic syndrome in Ghanaian postmenopausal women (Tables 9). The cut-offs values of the markers to predict the syndrome in Ghanaian postmenopausal women are 80.5 cm, 0.84, 0.61 and 0.34 for WC, WHR, HDL-C/TC and TG/HDL-C respectively. This finding partially agrees with a similar study conducted among Chinese postmenopausal women by Ruan *et al*., [31] which identified cut-off for WC to be 80.75 cm. This study also partially agrees with the IDF and WHO recommended WC and WHR cut-off points for European women (80 cm, 88 cm and 0.85 respectively) and other Eastern Mediterranean countries [32,33]. Similarly, WC cut-off points of 72, 82, 85, 86 and 88 cm provided the highest sensitivity for identifying hypertension in Nigerian, Cameroonian, Jamaican, St Lucian and Barbadians women respectively [34]. Even though BMI and WHtR had been explored to predict metabolic syndrome in several studies [31,35,36] , in this present study, the ROC analyses showed that BMI and WHtR could not be used to predict the presence of syndrome among Ghanaian postmenopausal women (Table 5). In general women in Ghana are defined as being overweight with BMI of 25 kg/ m² according to WHO criterion [37] but with a cut-off point of 23 kg/m² identified in both groups, there is the possibility that Ghanaian women develop metabolic syndrome at a lower anthropometric indices than the western populations. The accuracy of anthropometric variables as indicators of the syndrome was not high, as Swets [38] had postulated that 0.5 > AUC < 0.7 is an indication of the diagnostic being less accurate when ROC curves are applied in the diagnosis of conditions.

The use of TG/HDL-C and HDL-C/TC ratios to predict the presence of the syndrome had not been studied in Ghana. These ratios were able to predict the presence of the syndrome in Ghanaian postmenopausal women in this study. Since visceral adiposity is associated with hypertriglyceridemia, reduced HDL-C as well as insulin resistance, there is the likelihood that TG/HDL-C and HDL-C/TC ratios play important role in the pathogenesis of the syndrome and atherosclerosis. Plasma TG, TC and HDL-C are inversely related [39]. The enzyme Cholesterol-Ester Transfer Protein (CETP) balances the levels of TG and HDL-C, hence responsible for the joint exchange of TG and cholesterol ester between apoB-containing lipoproteins (chylomicrons, VLDL and LDL) and HDL. It has been postulated that high CETP activity explains some of the high TG levels and low HDL-C levels as witnessed in women with MetS [39].

Both obesity and atherogenic markers influence traditional metabolic risk factors in Ghanaian women. Liu *et al.,*[35] observed higher BMI, WC and WHtR values in Chinese women with high blood pressure, fasting blood glucose and triglyceride. Visceral abdominal fat had been recognized to predict insulin resistance and the presence of related metabolic abnormalities through overexposure of liver to free fatty acids [40-44]. Body composition changes occur in women mostly after menopause due to decrease secretion of oestrogen [22], resulting to age-related increases in obesity as well as metabolic disturbances [45]. In the present study, small WTR values were related to high FBG and TG among postmenopausal women (Table 2). This implies that Ghanaian postmenopausal women with smaller waist and larger thigh circumferences are at high risk of metabolic syndrome. Contrary, Snijder *et al*, [46] identified the association of lower risk of diabetes with larger thigh circumference among European women. Ryan *et al*., [47] also showed that African-American postmenopausal women had 34% greater midthigh low-density lean tissue area (a marker of intramuscular lipid content) than Caucasian postmenopausal women. The reason for the observation in Ghanaian postmenopausal women may be due to physical inactivity which could result in decrease and increase in muscle mass and visceral fat accumulation respectively in their thighs. Despite paucity of Ghanaian studies on physical activity or inactivity and its relation to obesity, evidence of physical inactivity is obtained from the growing problem of overweight (12.7%) and obesity (25.3%) especially among non-pregnant women aged 15–49 years [48]. Visceral fat in thighs can affect the activity of lipoprotein lipase resulting in increase in exposure of muscles to free fatty acids through uptake and storage. One of the sites responsible for insulin resistance is muscle mass [46]. The ratios TG/HDL-C and HDL-C/TC are associated with thigh circumference and WHR among postmenopausal women in this study (Table 3). This finding buttresses the point that the TG/HDL-C and HDL-C/TC can be explored as diagnostic tool for metabolic syndrome as well as atherosclerosis. In order to decrease the risk of metabolic syndrome and atherosclerosis among premenopausal and postmenopausal Ghanaian women, in general, life style modification to control weight, lipid profile, blood pressure and blood glucose should be emphasized.

## Conclusion

The present study suggested that WC, WHR, WHtR, TG/HDL-C and HDL-C/TC values were all associated with traditional metabolic risk factors. Waist-to-thigh ratio associated with raised glucose and triglyceride values. In Ghanaian postmenopausal women, waist circumference, WHR, TG/HDL-C and HDL-C/TC predicted the presence of metabolic syndrome.

## Abbreviations

MetS: Metabolic syndrome; H_MS: Harmonization; BMI: Body mass index; WHR: Waist-to-hip ratio; WC: Waist circumference; WTR: Waist-to-thigh ratio; WHtR: Waist-to-height ratio; SBP: Systolic blood pressure; DBP: Diastolic blood pressure; TC: Total cholesterol; TG, Triglyceride; HDL-C, High density lipoprotein cholesterol; LDL-C: Low density lipoprotein cholesterol; VLDL-C: Very low density lipoprotein cholesterol; HDL-C/TC: High density lipoprotein cholesterol-total cholesterol ratio; TG/HDL-C: Triglyceride-high density lipoprotein cholesterol ratio; CETP: Cholesterol ester transport protein.

## Competing interest

The authors declare that they have no competing interests.

## Authors’ contribution

FKNA designed the study and participated in drafting manuscript and result analysis. MA-F performed the sample collection, processed the data, as well as conducted statistical analysis and drafted the manuscript. FOM, JO-Y and LO participated in the design of the study and helped in analyzing data and in drafting the manuscript. All authors have read and approved the final manuscript.
